# Wind conditions shape co-migration fidelity in trans-Saharan birds at a Mediterranean stopover site

**DOI:** 10.1098/rsos.260539

**Published:** 2026-10-07

**Authors:** Erica Calabretta, Bruno Bellisario, Claudio Carere, Ivan Maggini, Massimiliano Cardinale, Elizabeth Yohannes, Leonida Fusani

**Affiliations:** ^1^ Department of Interdisciplinary Life Sciences, KLIVV, University of Veterinary Medicine, Vienna, Austria; ^2^ Konrad-Lorenz-Institut fur Evolutions und Kognitionsforschung, Klosterneuburg, Austria; ^3^ Department of Ecological and Biological Sciences, University of Tuscia, Viterbo, Italy; ^4^ Department of Aquatic Resources, Swedish University of Agricultural Sciences, Lysekil, Sweden; ^5^ Department of Bird Migration, Swiss Ornithological Institute, Sempach, Switzerland; ^6^ Department of Behavioural and Cognitive Biology, University of Vienna, Vienna, Austria

**Keywords:** ecological barrier, migration, North Atlantic Oscillation (NAO), temperature, tailwind, network modularity, co-occurrence networks, avian community

## Abstract

Weather is a key driver of bird migration, particularly near ecological barriers, where migratory species often converge before crossing. While its effects on individual timing are well documented, how weather reshapes the temporal assemblage of migratory bird communities remains unexplored. Using 18 years of standardised ringing data from 24 trans-Saharan species during pre-breeding migration, this study investigated whether weather conditions along the Mediterranean Sea influence annual network modularity, that is, the yearly interspecific clusters of co-occurrence at a stopover site, and measured the ‘co-migration fidelity’, defined as the tendency of species to co-occur in the same cluster over the years. Specifically, we examined the ‘modularity’ associations with tailwinds, temperature, and the North Atlantic Oscillation (NAO) index. Modularity increased significantly over time, revealing non-random species clusters whose arrival timing aligns under unfavourable winds. This suggests that less supportive winds may lead distinct species to migrate within the same narrow temporal window of favourable conditions, heightening co-migration fidelity. Conversely, modularity decreased sharply in years with stronger tailwinds, while temperature and NAO showed negligible effects. These findings provide novel evidence that shared environmental constraints during the sea crossing can shape the formation of migratory communities by prompting species' temporal convergence at a stopover.

## 1. Introduction

Bird migration is an outstanding natural phenomenon in which millions of individuals move simultaneously across continents, deserts, and oceans in response to shifting environments and resources [1]. Constrained by similar abiotic and biotic factors, such as the presence of major ecological barriers and internal regulatory mechanisms [2,3], many species seasonally overlap along the migratory route in the so-called ‘co-migration’ [4]. These coincidental assemblages of multiple species at critical stopover sites and migratory bottlenecks have been known for a long time, but only recently examined. DeSimone *et al.* [5] discovered the existence of persistent networked communities along the eastern North American coast, where non-random and positive interspecific interactions occur across a wide range of migratory species. However, the study did not directly assess whether these relationships arise as collective responses to changing environmental cues. Likewise, other works have examined the environmental drivers of migratory systems at the individual [6] or population levels [7], without adopting a community-based approach [8].

In recent decades, research on migration ecology has explored the role of non-breeding ecological conditions in the timing and success of pre-breeding migration. Variation in the quality and quantity of food sources at wintering areas, as a consequence of climate conditions, can affect departure timing [9–11]. Local weather conditions, such as the presence of tailwinds, also serve as essential cues to depart, particularly before crossing water barriers [12–15]. Indeed, Smith *et al*. documented that the overlap of different passerine species at coastal sites was strongly associated with wind conditions [16]. Similarly, warmer night-time temperatures can facilitate flight for nocturnal migrants [17,18]. In addition to local cues, large-scale climate indices, such as the North Atlantic Oscillation (NAO), can affect migration timing. Positive NAO phases can advance bird arrivals at central and northern latitudes because of improved environmental conditions [19–22] or delay the arrival of trans-Saharan species in a Mediterranean stopover site, while advancing them in Scandinavia [23]. Overall, ecological conditions at non-breeding and passage areas can shape the migratory journey at both the individual and community levels [24–27].

The island of Ponza is a strategic stopover site for a large number of migratory birds [28], offering a safe shelter after a long non-stop flight across the Mediterranean Sea [29–31]. A recent study [32] showed that African–European landbirds arriving on the island consistently migrate and arrive in well-defined groups of species, unveiling higher ‘fidelity’ in the pattern of co-occurrence (i.e. co-migration fidelity) in trans-Saharan migrants (i.e. birds that spend the non-breeding season south of the Sahara Desert) compared to species spending the non-breeding season in North Africa (i.e. short-distance migrants). The reduced availability of optimal migration conditions over time may prompt species to co-occur during their travel, especially in landbirds for which crossing water barriers without a chance to rest represents a significant challenge [14,33]. Yet, how environmental conditions at non-breeding areas and over ecological barriers lead to the temporal formation of migratory bird communities remains poorly understood.

In this study, we investigated whether and how weather conditions along the sea route are associated with the temporal clustering of co-occurring species at the stopover site of Ponza during the pre-breeding migration. Using 18 years of ringing data (2007–2024) from 24 trans-Saharan species, we assessed the influence of NAO, tailwind, and temperature along the Mediterranean route on the annual co-occurrence network and measured the co-migration fidelity over the years. We hypothesized that negative NAO values and less favourable weather (headwinds or lower temperatures) at sea would induce simultaneous passage and temporal clustering of co-occurring species on the stopover island of Ponza, resulting in high co-migration fidelity across species.

## 2. Material and methods

### 2.1. Study site and bird ringing data

The study was carried out on the island of Ponza, about 50 km off the western Italian coast (40°55′ N, 12°58′ E). Located along one of the main central Mediterranean migratory corridors, Ponza is an important stopover site for large numbers of migratory birds of several species. The ringing station has been active every year since 2002 during the pre-breeding migratory season (March–May). Birds were captured daily using mist nets that were checked every hour from sunrise to sunset [28,34]. As nets were located on slopes with different orientations, a subset of nets could be kept open even with moderately strong winds, and only on days with predominantly rain or too strong winds, all nets were kept closed. Consequently, adverse meteorological conditions prevented catching birds for only 1.55% of the total study period. We examined long-term ringing data for 24 trans-Saharan species. Specifically, we included 349 802 individuals caught between March and May from 2007 to 2024, the period during which the sampling procedures were fully standardized by daily effort.

### 2.2. Climate data

To assess the effects of climate conditions on co-migration fidelity, we examined the NAO and weather at sea. The NAO index was collected monthly from the NCEP/NCAR R1 dataset (Boulder, CO, USA; https://psl.noaa.gov/data/climateindices/list/#NAO) to measure its seasonal mean from March to May each year. This enabled the capture of general atmospheric conditions during the migratory window. Using the ‘RNCEP’ package (v1.0.11; [35] of R v4.2.1; [36]), we extracted weather data from the Reanalysis II project [37]; https://psl.noaa.gov/about/, for the central Mediterranean (40°0′ N, 12°30′ E). This location was chosen assuming that migratory birds arriving on Ponza followed the shortest sea-crossing route from the closest North African coast (Tunisia; [38,39]).

Passerine migrants typically crossed large water barriers by departing after dusk and travelling overnight [33,40,41], favouring altitudes around 750 m above sea level when flying over the Mediterranean Sea [40,42]. Accordingly, we analysed air temperature and tailwind support during the night (from 00.00 to 06.00) before the birds' arrival on Ponza at three different pressure levels: sea surface (1000 mb, i.e. millibar), approximately 700 m elevation (925 mb), and approximately 1500 m elevation (850 mb). To calculate the tailwind component, we estimated the birds' direction by computing the loxodrome angle between the assumed departure site in Tunisia (37°01′ N, 11°00′ E) and Ponza (direction = 21.37°). We then quantified flow assistance using the equation *fa*_tailwind_ = *y*cos*ϴ* [35], where *fa*_tailwind_ is tailwind flight assistance, *y* is the wind speed (m s^−1^), and *ϴ* is the angular difference between the direction of the bird's movement and the wind.

Weather variables were selected for the migratory period between March and May from 1993 to 2024. To assess the seasonal interannual variability of air temperature and tailwind within a consistent climatological framework, we first calculated the seasonal mean of each variable for every year. Then, we measured the climate normal (CN), defined as the 30 year average of each weather variable [43], using the fixed reference period 1993–2023. Finally, anomalies in air temperatures and tailwinds were derived by subtracting the CN from each year's seasonal mean. This allowed us to quantify temperature and tailwind anomalies for our study period (March–May over the 2007–2024 migratory season) relative to the long-term climatological normal.

### 2.3. Network inference and co-migration fidelity

To identify which species tended to arrive at the stopover site on the same day, we inferred yearly co-occurrence networks using Spearman rank correlations of species' daily abundances (i.e. number of individuals captured daily per species). We accounted for temporal autocorrelation using lag-1 partial correlations, which isolated the relationship between species by controlling for their own abundance on the previous day [32,44]. To ensure robust network inference, we applied a quality-control filter to exclude species with extremely low annual abundances (< 2.5th percentile of the abundance distribution for that year). Since our 24 focal trans-Saharan species were consistently abundant at Ponza, all species exceeded this threshold across the entire study period (2007–2024) and were thus retained for analysis. In the built unweighted networks, the links (i.e. co-occurrence) between nodes (i.e. species) represented a significant and positive covariation (*r* > 0.5 and *p* < 0.05) between their temporal abundances, indicating a high probability of co-occurrence among species. Additionally, we constructed unweighted networks based on negative associations (*r* < −0.5 and *p* < 0.05) where the links between species represented temporal segregation in their occurrence patterns. In this way, we could measure both the degree of association and dissociation across species.

We then quantified modularity (*Q*), a measure of the extent to which networks are subdivided into modules (aka clusters), that is, groups of highly connected nodes with relatively few connections between groups [45]. We used the Louvain algorithm in the package ‘igraph’ of R v2.1.3; [46], which identified communities of densely connected nodes by iteratively optimizing *Q* through a hierarchical, modularity-maximizing process [45]. Higher modularity values indicated a stronger tendency of networks to partition into modules of species with common migration timing [32]. For the networks based on negative associations, higher modularity values corresponded to species that never co-occurred at the stopover site.

To evaluate whether the observed modularity differed from random expectations owing to species abundance distributions or sampling effects, we generated 1000 null networks for each year by randomly rewiring the network while preserving the degree sequence (i.e. the number of associations of each species) [47]. Modularity was calculated for each null network using the same Louvain algorithm applied to the observed networks. For comparability, both observed and null modularity time series were smoothed using a simple moving average (SMA) with automated window selection based on the Akaike information criterion [48], implemented via the ‘smooth’ package (v4.1.0; [49]). We then calculated the mean and 95% confidence interval of null modularity across simulations, and observed modularity values exceeding this interval were interpreted as evidence of statistically significant modular structure. After that, to test for a temporal trend in modularity, we applied the Mann–Kendall statistics to the smoothed observed modularity time series using the ‘trend’ package (v1.1.6.; [50]).

We assessed whether temporal variations in raw network modularity were independent of potential structural network constraints across years by calculating standardised modularity (*zQ*) for each year as: *zQ* = (*Q*_observed_ – *Q*_null_)/*σQ*_null_, where *Q*_null_ and *σQ*_null_ are the mean and standard deviation of modularity across the 1000 null networks for that year. Because annual weather conditions might directly shape species co-occurrence patterns, and thus the network's degree structure, standardising modularity (*Q*) against a degree-preserving null model risked removing the very environmental signal being tested [47]. We therefore used the observed modularity (*Q*) as our primary response variable, whereas the standardised modularity score (*zQ*) was used only as a robustness check to confirm that the observed long-term trend was not an artefact of network topology (supplementary material, figure S1).

Using the normalized mutual information (nmi; [51]), we measured the ‘co-migration fidelity’, defined as the frequency with which two species tended to co-occur in the same module over the years [32]. Modularity partitions obtained from each year's network were used as input, where each species was assigned to a module representing its migration grouping for that year. The nmi is a robust metric for comparing different partitions (community modular subdivision) of the same network, measuring the shared information between two partitions and providing a similarity score between 0 (independent) and 1 (identical). Here, a nmi value of 1 indicated perfect convergence in time, meaning that two species always co-occurred in the same module over the years; instead, a value of 0 reflected complete independence, hence the two species never co-occurred within the same module across years. Data were organized into a matrix with species in the rows and year in the columns to calculate the pairwise nmi values, providing a symmetric similarity matrix showing the co-migration fidelity across species (supplementary material, table S1).

### 2.4. Data analysis

To investigate which environmental factors might shape changes in species grouping over time, we performed generalized additive models (GAM) for each pressure level (1000, 925, and 850 mb) using the R package ‘mgcv’ (v1.9-1; [52]) to accommodate potential non-linear relationships. In each model, *Q* was the response variable, while the seasonal mean of NAO, the anomalies in tailwinds and temperature were the environmental predictors. To account for potential effects of NAO on weather conditions, we also examined the interaction between the anomalies of each weather variable and the seasonal NAO in each model. Using the R packages ‘performance’ (v0.13.0; [53]), ‘mgcv’ [52], and ‘mgcViz’ (v0.2.0; [54]), we performed diagnostic tests on all models and verified the homogeneity of variance by plotting residuals versus fitted values using the R package ‘gratia’ (v0.9.2; [55]).

## 3. Results

### 3.1. Modularity

Although there were fluctuations in the initial period with low *Q* values, the extent of temporal co-occurrence network modularity increased significantly throughout the years (Mann–Kendall trend test *z* = 2.1212 and *p* = 0.0339). *Q* ranged from the lowest value of 0.229 in 2014 to the highest one of 0.240 in 2023, corresponding to more than three standard deviations of the interannual variability, with an average *Q* (± s.d.) of 0.235 ± 0.003 (figure 1). Observed *Q* values also significantly and consistently exceeded the expectations from the null model (figure 2). This long-term upward trajectory was further confirmed when using standardised modularity scores (*zQ*), demonstrating that the increase in compartmentalization is robust to degree sequence constraints (Mann–Kendall test, *z* = 4.62, *p* < 0.000003; supplementary material, figure S1). By contrast, *Q* of species dissociation showed a significant decreasing trend over time (Mann–Kendall test, *z* = −4.85, *p* < 0.001; supplementary material, figure S2).

**Figure 1.**
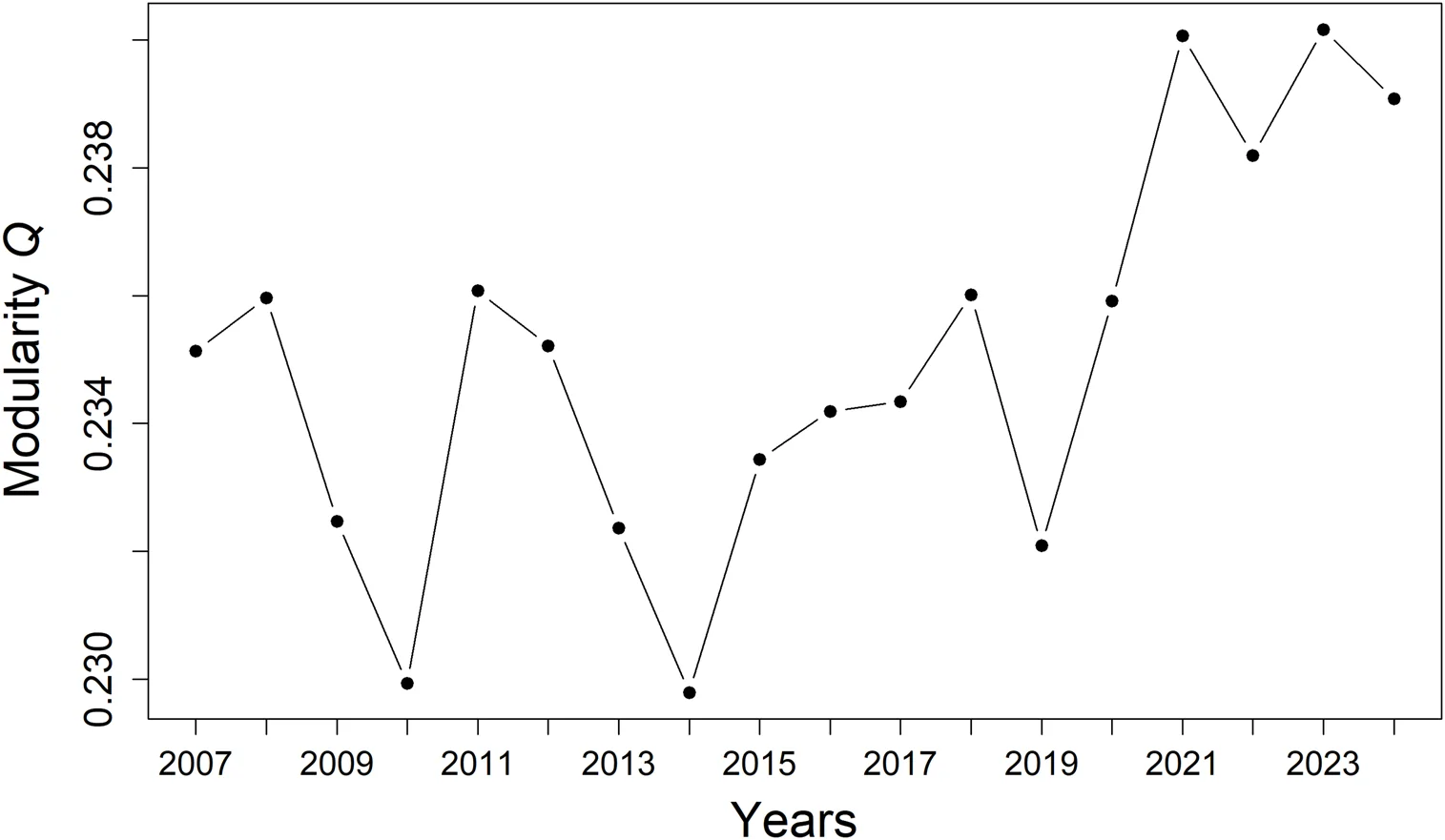
Temporal trend of modularity (*Q*), showing the extent to which co-occurrence networks subdivide into groups of species characterized by common migration timing. The solid black line connects the time series (2007–2024), where dots show *Q* for each year.

**Figure 2.**
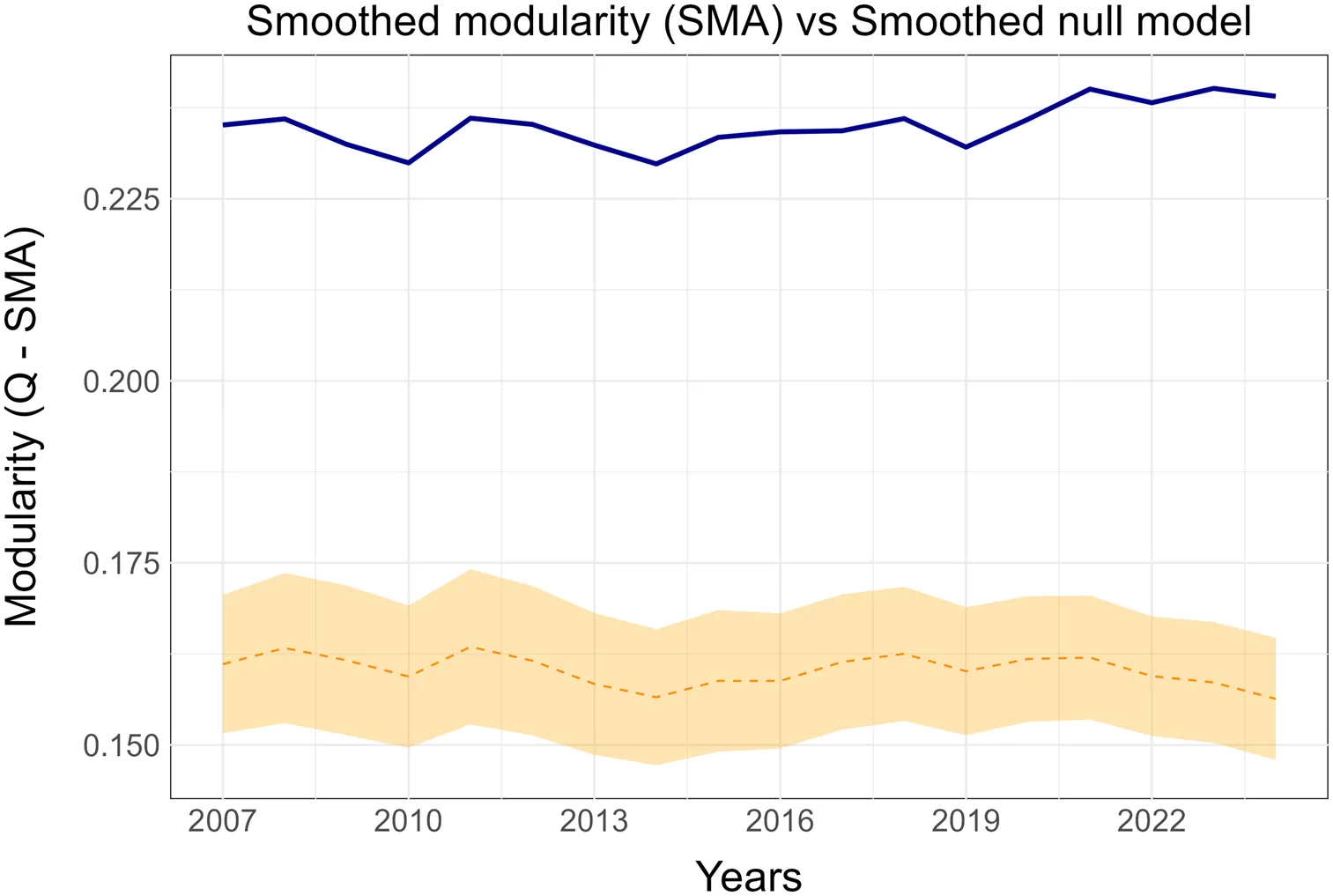
Smoothed modularity (*Q*) of the observed data (dark blue line) compared to the smoothed modularity derived from the null models from 2007 to 2024. The dashed orange line represents the mean modularity of the null model, and the shaded orange ribbon shows the 95% confidence interval of the null model after smoothing.

### 3.2. Co-migration fidelity

The pairwise co-migration fidelity identified eight main clusters across the 24 trans-Saharan species (cut = 0.52; figure 3). Among them, six clusters comprised more than one species, with three of them displaying high fidelity values (dissimilarity < 0.4; similarity values in the supplementary material, table S2), and three other clusters showing moderate fidelity values (dissimilarity < 0.55); while two clusters comprised only one species, thereby showing no association.

**Figure 3.**
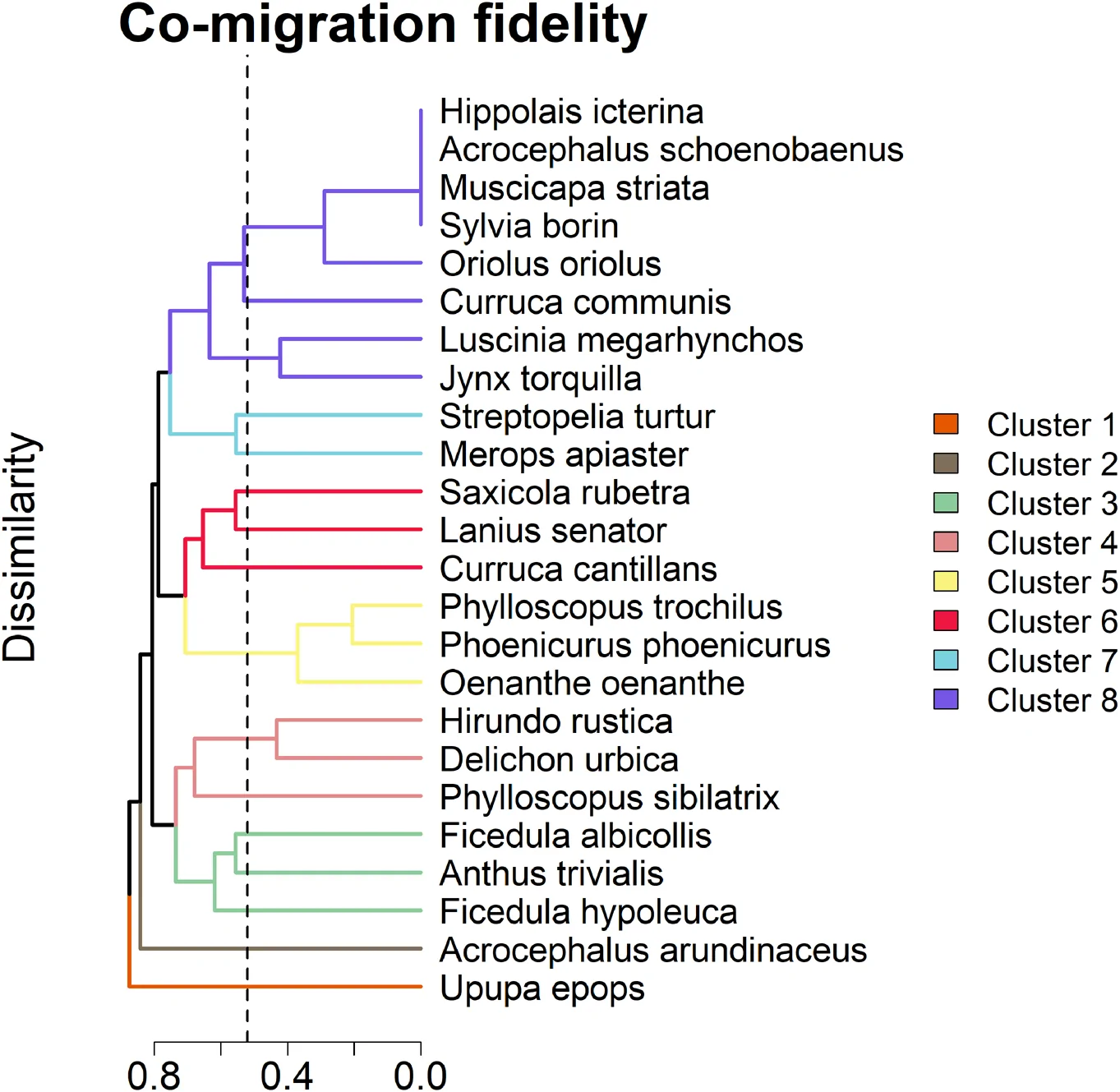
Dendrogram of co-migration fidelity among 24 trans-Saharan species. Dissimilarity indicates the frequency of their co-occurrence within the same module across years (0 = complete temporal convergence; 1 complete temporal divergence). Hierarchical clustering was performed using average linkage, with colour highlighting the resulting species clusters.

### 3.3. Climatic patterns

Modularity (*Q*) was significantly correlated with tailwind anomalies at 1000 mb (figure 4; table 1), showing a similar, non-significant trend at 925 mb but not at 850 mb (supplementary material, figures S3 and S5). On the other hand, *Q* was not correlated with temperature anomalies at any pressure level (figure 5; table 1; supplementary material, figures S4 and S6 at 1000, 925, and 850 mb, respectively). Neither seasonal NAO nor its interaction with anomalies in temperature and tailwind was significantly correlated with *Q* at any pressure level.

**Figure 4.**
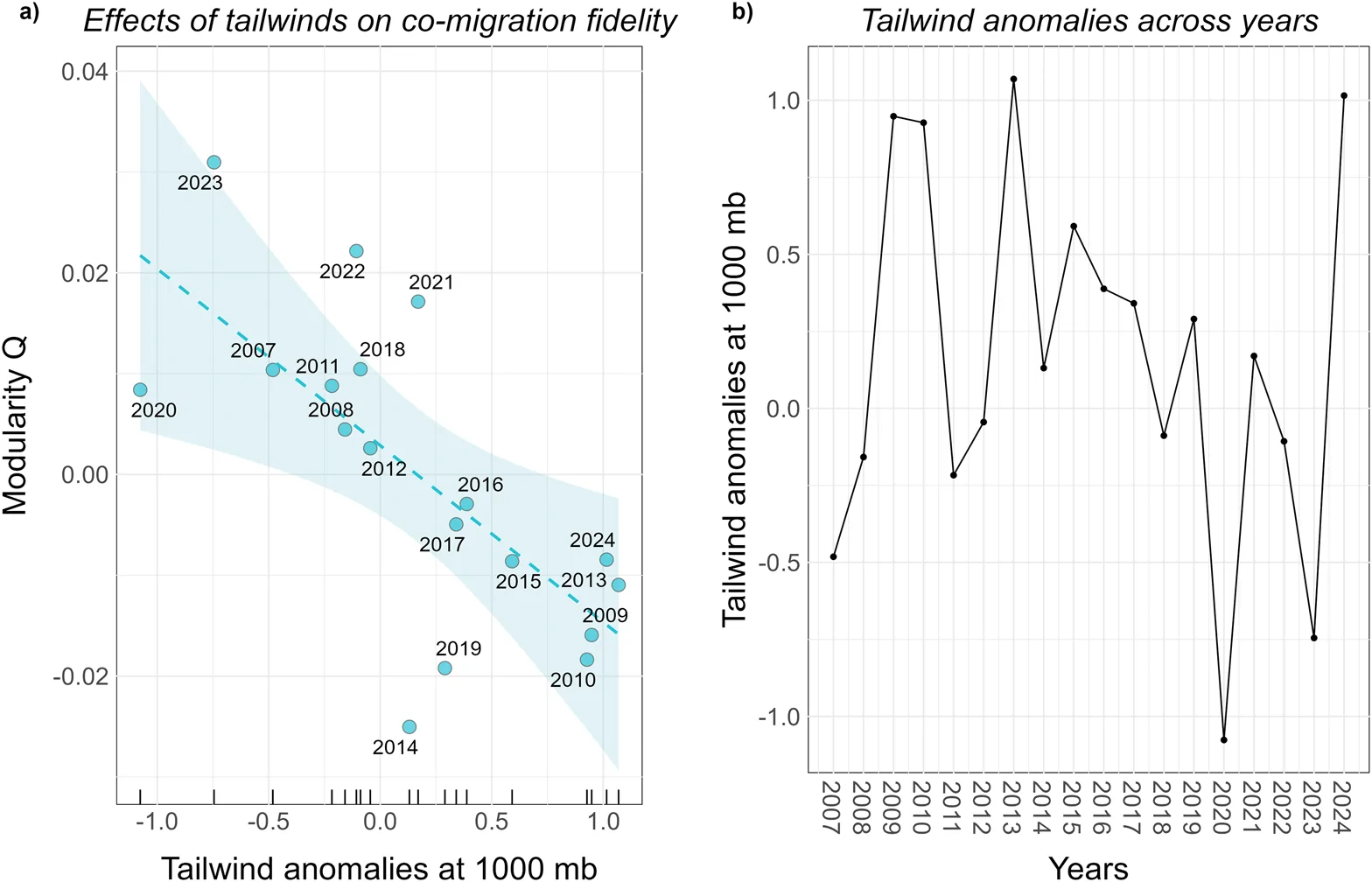
(a) Network modularity (*Q*; partial residuals) as a function of tailwind anomalies along the Mediterranean route to Ponza during the night before the bird's arrival. Each circle indicates the corresponding year. The blue dashed line shows the fitted values with the 95% confidence intervals (light blue area). (b) Tailwind anomalies measured at 1000 mb (approx. 100 m.a.s.l.) over the Mediterranean route across the study period (2007–2024).

**Table 1.** Results from general additive models (GAM) assessing the effects of seasonal NAO, anomalies in tailwind and air temperature, and their respective interaction with NAO at each pressure level (1000 = approx. 100 m.a.s.l.; 925 = approx. 700 m.a.s.l.; 850 = approx. 1500 m.a.s.l.) on network modularity (*Q*). (Significant *p*-values refer to the smooth term significance and are highlighted in bold. ‘Mean effect’ indicates the average predicted response on the link scale for each focal predictor, with all other predictors kept at their mean. The width of the confidence interval (CI) represents the average 95% CI span. Variation inflation factor (VIF) values indicate the degree of multicollinearity among predictor variables.)

pressure levels	predictors/ metrics	anomalies temperature	anomalies tailwind	seasonal NAO	anomalies temperature approximately NAO	anomalies tailwind approximately NAO
**1000**	*p*-value	0.153	**0.008**	0.326	0.122	0.521
	mean effect	−1.178	−1.182	−1.185	−1.177	−1.182
	95% CI width	0.025	0.025	0.026	0.038	0.038
	VIF	1.87	1.57	1		
**925**	*p*-value	0.698	0.252	0.539	0.458	0.572
	mean effect	−1.179	−1.18	−1.179	−1.179	−1.18
	95% CI width	0.03	0.029	0.031	0.048	0.054
	VIF	1.05	1.6	1		
**850**	*p*-value	0.464	0.916	0.272	0.335	0.076
	mean effect	−1.178	−1.179	−1.173	−1.172	−1.172
	95% CI width	0.034	0.036	0.029	0.048	0.047
	VIF	1.44	3.91	1

**Figure 5.**
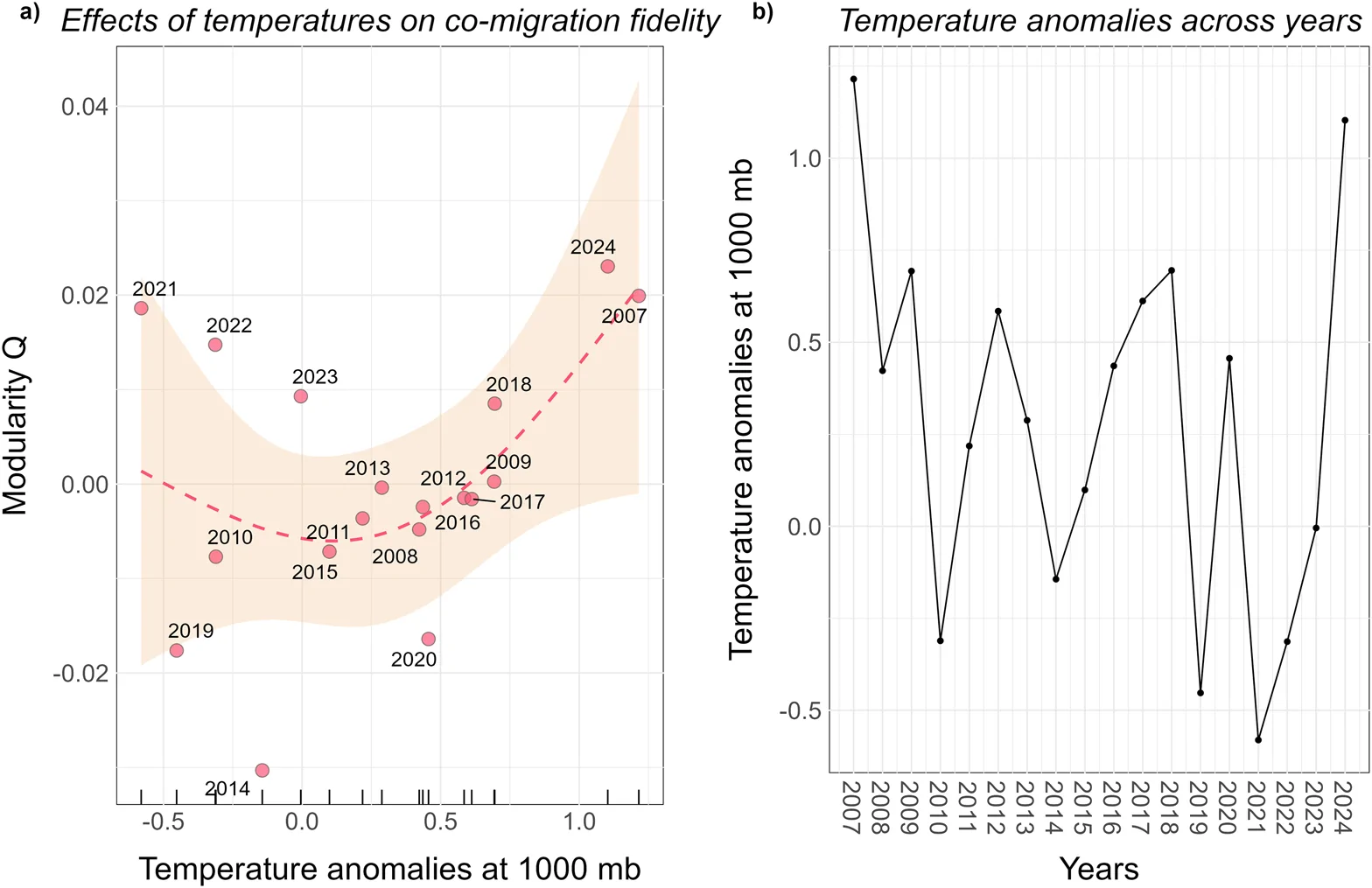
(a) Network modularity (*Q*; partial residuals) as a function of temperature anomalies along the Mediterranean route to Ponza during the night before the bird's arrival. Each circle indicates the corresponding year. The red dashed line shows the fitted values with the 95% confidence intervals (orange area). (b) Temperature anomalies measured at 1000 mb (approx. 100 m.a.s.l.) over the Mediterranean route across the study period (2007–2024).

## 4. Discussion

Our findings show that weather conditions during the Mediterranean crossing are associated with the temporal clustering of co-occurring trans-Saharan species at the pre-breeding stopover site. As predicted, the annual network modularity revealed non-random and defined clusters of species with shared migration timing, extending the patterns of co-migration fidelity detected in the previous study of Bellisario *et al.* [32]. Across the nearly 20 years analysed, modularity increased and showed a consistent and significant correlation with tailwind anomalies along the Mediterranean Sea. Specifically, less supportive winds were associated with tighter temporal alignment of species modules at the stopover site, indicating that interannual variation in wind conditions influences the extent of network partitioning. Different from our predictions, temperature anomalies, the seasonal mean NAO and its interactions with weather variables were not significantly associated with modularity. Our results, therefore, suggest that tailwind anomalies not only shift the timing of individual species but may also act as a common atmospheric filter under which migratory birds exhibit temporally cohesive groups.

### 4.1. Climatic patterns

In line with our predictions, tailwind anomalies during the sea crossing influenced the tendency of species to subdivide into clusters with common migration times over the years. This underpins previous studies which found that the selection of favourable winds had a greater impact than other meteorological factors to face the endurance flight over the Mediterranean Sea [56–59]. It also aligns with other studies showing that supportive winds are associated with the assemblage of multiple species at coastal sites [16], as well as departure decisions at the edge of a water barrier crossing, regardless of the species involved [15]. The effect of tailwind anomalies was detected at the lowest atmospheric level (1000 mb), showing a similar, non-significant trend around 700 m.a.s.l., but not at higher altitudes (850 mb). This suggests that birds assess weather conditions primarily near the sea surface, which may be more relevant for take-off and flight over the Mediterranean Sea [42]. As supportive winds generally facilitate migratory flights by reducing travel time and costs [60], less favourable conditions may prompt individuals of different species to delay departure and gather at the coastline before crossing the sea. When a suitable wind finally occurs, these individuals may depart at the same time, consequently aligning their migratory schedules and their co-occurrence on the stopover island. However, this proposed mechanism represents one plausible interpretation of the observed pattern. Alternatively, persistent unfavourable weather could induce broad-scale temporal overlap across a wider range of species rather than within distinct subgroups. Such a generalized response might increase the network's overall connectivity and reduce its modularity by blurring the boundaries between modules relating to different species. Distinguishing between these two scenarios will require detailed future analyses examining interannual variation in node degree and network density alongside modularity.

Contrary to our predictions, anomalies in air temperature during nocturnal flights over the sea did not significantly affect the yearly organization of species in migration modules. Across the different atmospheric levels, the relationship between temperature anomalies and modularity was weak and highly variable, and thus not sufficiently strong to explain the clustering patterns. This outcome is partly consistent with earlier findings where tailwinds along the Mediterranean led to the advancement of pre-breeding migration in several species, while higher temperatures played a major role in Central Europe [61]. Similarly, a radar study on nocturnal bird migration over the North Sea found that temperature differences were less influential than tailwinds and high atmospheric pressure during nights of intense migration [62].

Moreover, neither the seasonal mean of NAO nor its interactions with tailwind and temperature anomalies had effects on the temporal clustering of co-occurring species on Ponza over the years. Also, earlier studies in several trans-Saharan migrants documented no influence of NAO on their pre-breeding migration timing, either along the migratory path [26,63,64] or at the breeding areas [19], as well as on their survival rates [65]. On the other hand, other studies highlighted the need to combine the analysis of multiple large-scale climate indices to evaluate their potential effects on pre-breeding migration [66,67]. Still, the lack of significance of even the interactions between the NAO and local weather conditions suggests that its role in the Mediterranean region might remain more marginal than in non-breeding sites or northern latitudes [68].

Overall, these results emphasise that wind conditions are the main climatic variable associated with annual network modularity across species during the pre-breeding migration over the Mediterranean Sea. Broader climate analyses over the Mediterranean basin showed a significant decadal variability in wind regimes [69]. While wind variability is known to shift departure and flight timing in individual species [33], our findings demonstrate how these responses correspond to temporal assemblage and species clustering at the community level under shared, less supportive winds.

### 4.2. Co-occurrence at a stopover site

Beyond climatic influences, we also explored how community structure emerged at the stopover island. Contrary to former findings [32], yearly co-occurrence networks in this study did not exhibit high modularity values. The range of yearly network inferences points to a relatively weak community structure (*Q* = 0.229–0.240; [69]). This discrepancy probably results from the number of species considered since this study focused only on trans-Saharan bird species and excluded short-distance migrants. Nonetheless, although the absolute variation in *Q* was numerically small, it was substantial when compared to the low interannual variability (s.d. = 0.003), and modularity was consistently higher than expected under random association. In parallel, the significant decrease in species segregation over time indicates that avoidance patterns became less pronounced. Rather than exhibiting partitioned transit schedules, species appear increasingly subjected to shared environmental filters that passively aggregate them into the same arrival windows. Consequently, this reduction in interspecific segregation directly underpins tighter clustering of co-occurring species at the stopover site.

Changes in migration phenology could also influence the rise of migratory communities during the Mediterranean crossing. As the timing of many species has advanced over recent decades because of climate change, while others have shown a weak trend [23,70], it is possible that such shifts lead to an apparent increase in species' temporal co-occurrence. However, previous research at our study site has shown that the total duration of the migratory passage for most species has broadened, rather than contracted, over the last two decades [71]. Because the seasonal window is expanding, the increasing modularity we observe is unlikely to be an artefact of a shortened migration period. Instead, it suggests that external environmental factors, such as the narrowing of optimal wind windows, may override wider phenological expansions, resulting in restricted arrival windows at the stopover.

Even though the significant annual co-occurrence network cannot be used as a proxy for ecological interactions [72], shared temporal passage may still foster positive relationships between species both in flight and upon arrival at stopover sites [5]. For instance, nocturnal migrants with analogous flight calls and wing morphologies, but not closely related phylogenetically, tend to stick together [73], particularly when crossing a water barrier [74]. Acoustic similarity has also been shown to reflect overlapping migration schedules among phylogenetically related species [75] and to aid the estimation of stopover quality in species with similar habitat ecology [76]. Indeed, temporal assemblage could optimize the costs of the migratory journey, increasing the chances of survival by facilitating navigation and sharing important information in flight [4]. However, the likelihood of modular organization at a stopover site may be reduced by interspecific competition [77,78] and by different migration strategies in relation to the predation risk [79]. Species co-occurrence within migration networks may arise from multiple processes, including shared migratory routes and environmental constraints, rather than indicating direct associations among species. In this study, we focused on network modularity because it depicts the emergent organization of the community by identifying groups of species that consistently co-occur across years. Additional topological descriptors, such as average node degree or the distribution of pairwise association strengths, could complement this perspective by describing variation in local connectivity patterns [80]. However, these metrics capture different aspects of network structure: changes in species-level connectivity do not necessarily translate into changes in community-level modular organization [81]. Future studies integrating species-level association dynamics with higher-order network properties will provide a more comprehensive understanding of how individual associations scale up to shape migratory communities under changing climatic conditions.

Altogether, these findings indicate that, despite absolute modularity values remaining relatively low, over time species were increasingly organized into temporally cohesive and non-random groups, regardless of species' abundance distribution or sampling effects. This pattern reflects a gradual and significant strengthening of community structure where different species are progressively exhibiting simultaneous passage across the Mediterranean Sea. However, our study does not allow us to draw conclusions on potential social interactions derived from the temporal assemblage. The observed intensification of modularity seems most likely a passive consequence of environmental forces that conduct species to overlap at stopover.

## 5. Conclusions

To our knowledge, this is the first study to provide empirical evidence that the formation of landbird communities is associated with tailwind anomalies over a large ecological barrier, resulting in non-random and distinct clusters of co-occurring species at a stopover site during pre-breeding migration. This phenomenon has intensified over the years, reflecting a long-term strengthening of co-migration fidelity that may arise from shifting species phenology or shared environmental pressures along the flyway.

The ongoing environmental and climate crisis is altering many aspects of wildlife migration [82,83]. In this context, temporal co-occurrence of species groups may represent a passive ecological response to facilitate the crossing of large barriers during strenuous flights, at the same time ensuring the arrival at stopovers and breeding grounds under changing atmospheric conditions. Still, it may also reinforce competition among species with similar ecological requirements which converge in the same environment [84]. The fact that Ponza offers limited refuel opportunities and is mainly used for short rests [34,85] further supports the idea that species assemblages primarily arise from shared environmental constraints at departure and along the sea crossing. Such interpretation is consistent with the observed decline in species avoidance patterns. Therefore, the increased temporal clustering structure of diverse landbird species owing to less supportive winds at sea sheds light on the need to extend the study of migration beyond single-species approaches and consider migratory communities as dynamic, climate-sensitive units. Future research would benefit from adopting a community-based approach to understand how ongoing environmental changes may alter migratory patterns across space and time, especially at critical stopover sites. Identifying the climatic and ecological factors that shape community-level migration will not only help anticipate the impacts of environmental conditions but will also guide conservation strategies to protect diverse migratory bird species, balancing ecological demands across different stages of the life cycle.

## Supplementary Material



## Data Availability

Data are provided on the Phaidra Digital Repository https://doi.org/10.34876/4gtc-yr28, and in the supplementary material [86].
