# Supplementary material for: Wind conditions shape co-migration fidelity in trans-Saharan birds at a Mediterranean stopover site
Source: R Soc Open Sci. 2026 Oct 7;13(10):260539. doi: 10.1098/rsos.260539 (PMC13641894; doi:10.1098/rsos.260539)
Supplement: Supplementary file 1 [file rsos.260539.zip › rsos.260539/rsos260539_si_001.docx]

**Wind conditions shape co-migration fidelity in trans-Saharan birds at a Mediterranean stopover site.**

*Erica Calabretta^1,2*^, Bruno Bellisario^3*^, Claudio Carere^3^, Ivan Maggini^1^, Massimiliano Cardinale^4^, Elizabeth Yohannes^5^, Leonida Fusani^1,6^*

*^1^ KLIVV, Department of Interdisciplinary Life Sciences, University of Veterinary Medicine, Vienna, Austria*

*^2^ KLI for Evolution and Cognition Research, Klosterneuburg, Austria*

*^3^ Department of Ecological and Biological Sciences, University of Tuscia, Viterbo, Italy*

*^4^ Department of Aquatic Resources, Swedish University of Agricultural Sciences, Lysekil, Sweden*

*^5^ Department of Bird Migration, Swiss Ornithological Institute, Sempach, Switzerland*

*^6^ Department of Behavioural and Cognitive Biology, University of Vienna, Austria*

Corresponding author: [leonida.fusani@univie.ac.at](mailto:leonida.fusani@univie.ac.at)

*Co-primary authors

Figure S1. Temporal trend of standardised modularity (zQ), controlling for annual network distributions. Values were calculated relative to 1,000 degree-preserving null networks per year : zQ= (Qobserved – Qnull)/σQnull. The solid blue line connects the time series (2007–2024), where dots show zQ for each year, confirming that the long-term increase in modularity remains consistent when controlling for local connectivity.

Figure S2. Temporal trend of modularity (Q), showing the extent to which networks subdivide into groups of species that never co-occur at the stopover site. The solid black line connects the time series (2007-2024), where dots show Q for each year.

Table S1. Symmetric similarity matrix measured using the pairwise normalised mutual information (nmi). The dark pink and the turquoise show the species with the highest co-migration fidelity (nmi > 0.70 and nmi = 1, respectively).

*Outputs of GAM for the other pressure levels*

Figure S3. Modularity (Q; partial residuals) as a function of the anomalies in tailwinds at 925 mb (~ 700 m asl) along the Mediterranean route to Ponza during the night before the bird’s arrival. Each circle represents the corresponding year. The blue dashed line shows the fitted values with the 95% confidence intervals (light blue area).

Figure S4. Modularity (Q; partial residuals) as a function of the anomalies in temperatures at 925 (~ 700 m asl) along the Mediterranean route to Ponza during the night before the bird’s arrival. Each circle represents the corresponding year. The red dashed line shows the fitted values with the 95% confidence intervals (orange area).

Figure S5. Modularity (Q; partial residuals) as a function of the anomalies in tailwinds at 850 (~ 1500 m asl) along the Mediterranean route to Ponza during the night before the bird’s arrival. Each circle represents the corresponding year. The blue dashed line shows the fitted values with the 95% confidence intervals (light blue area).

Figure S6. Modularity (Q; partial residuals) as a function of the anomalies in temperatures at 850 (~ 1500 m asl) along the Mediterranean route to Ponza during the night before the bird’s arrival. Each circle represents the corresponding year. The red dashed line shows the fitted values with the 95% confidence intervals (orange area).
